# Natural Antimicrobials as Additives for Edible Food Packaging Applications: A Review

**DOI:** 10.3390/foods10102282

**Published:** 2021-09-26

**Authors:** Sneh Punia Bangar, Vandana Chaudhary, Neha Thakur, Priyanka Kajla, Manoj Kumar, Monica Trif

**Affiliations:** 1 Department of Food, Nutrition and Packaging Sciences, Clemson University, Clemson, SC 29631, USA; 2 College of Dairy Science and Technology, Lala Lajpat Rai University of Veterinary and Animal Sciences, Hisar 125001, India; 3 Department of Livestock Product Technology, Lala Lajpat Rai University of Veterinary and Animal Sciences, Hisar 125001, India; nehathakur@luvas.edu.in; 4 Department of Food Technology, Guru Jambheshwar University of Science and Technology, Hisar 125001, India; kajlapriyanka@yahoo.com; 5 Chemical and Biochemical Processing Division, ICAR–Central Institute for Research on Cotton 10 Technology, Mumbai 400019, India; manoj.kumar13@icar.gov.in; 6 CENCIRA Agrofood Research and Innovation Centre, Research and Development Department, Ion Meșter, 6, 400650 Cluj-Napoca, Romania

**Keywords:** natural antimicrobial additives, edible films, essential oils, bacteriocins, bacteriophages, casting methods, food applications

## Abstract

Edible packaging is a swiftly emerging art of science in which edible biopolymers like lipids, polysaccharides, proteins, resins, etc., and other consumable constituents extracted from various non-conventional sources are used alone or imbibed together. Edible packaging with antimicrobial components had led to the development of the hypothesis of active packaging which safeguards the quality of foods as well as health of consumers. Natural antimicrobial agents (NAMAs) like essential oils from spices, bioactive compounds derived from vegetables and fruits, animal and microorganism derived compounds having antimicrobial properties can be potentially used in edible films as superior replcement for synthetic compounds, thus serving the purpose of quality and heath. Most of the natural antimicrobial agents enjoy GRAS status and are safer than their synthetic counterparts. This review focuses on updated literature on the sources, properties and potential applications of NAMAs in the food industry. This review also analyzes the biodegradability and biocompatibility and edibility properties of NAMAs enriched films and it can be concluded that NAMAs are better substitutes but affect the organoleptic as well as the mechanical properties of the films. Despite many advantages, the inclusion of NAMAs into the films needs to be investigated more to quantify the inhibitory concentration without affecting the properties of films and exerting potential antimicrobial action to ensure food safety.

## 1. Introduction

Antimicrobials play an important role in ensuring food safety in the food manufacturing and packaging industries. [1]. Additionally, the use of plastics in food packaging systems has led to an unprecedented increase in plastic pollution in the environment [2], which is projected to double globally by 2050 [3,4]. These two major challenges have led food technologists to develop the idea of antimicrobial films that beat the problem of both plastic pollution and antimicrobial resistance. The aim is to develop films that maintain food quality through various incorporated additives and ensure food safety by keeping the microbial count within the permissible limits [5]. Edible packing safeguards food products from physical, chemical and biological hazards, much like conventional synthetic polymer-based packing. However, the addition of natural antimicrobial agents (NAMAs) to the film formulation makes these biodegradable films active in nature [6], which serve the purpose of protection and preservation. Due to the overburdening of the environment by plastics, scientists are looking for sustainable and biopolymers to form edible films.

Several extracts derived from naturally occurring spices and herbs derived, plant-based secondary metabolites, essential oils and microorganisms can serve as a source of NAMAs to be used in edible films. A majority of these are still undiscovered and underutilized as an alternative to synthetic antimicrobials in food. They are not popular due to poor commercial adaptability, which is actively being researched and improved upon. Encapsulation of NAMAs in edible films can effectively check microbial growth in foods and enhance the keeping quality of food [7]. NAMAs are incorporated in matrixes formed by biopolymers like cellulose, chitosan, pectin, starch, gellan gum, xanthine, alginate, carrageenan, agar and kefiran for use in biodegradable packaging systems that are active [8,9]. NAMAs serve as bio-preservatives in active food packaging systems and ensure the good health of consumers. Most of the NAMAs enjoy GRAS status and are safer than their synthetic counterparts [10].

Additionally, certain NAMAs offer nutritional benefits as well. A problem of pungency and toxicity in the food product may result from the unchecked use of NAMAs in edible films. Hence, before using, their levels need to be standardized in the film formulation to give maximum protection with minimum toxicity and negligible flavor problems. Certain NAMAs also act as potent antioxidants preventing rancidity in certain foods. Furthermore, their addition affects certain properties like tensile strength, thickness, opacity, etc. [11].

Since the application of films and coatings, including natural agents, is expanding due to their biodegradability and potency to enhance food safety, quality and shelf life, this review is to develop and to understand NAMAs, their use in edible films/coatings, method of manufacture, research done so far and materials developed using NAMAs with a special focus on their benefits, limitations and future prospects in the field of active edible packaging.

## 2. Sources of NAMAs in Edible Films

**Figure 1:**
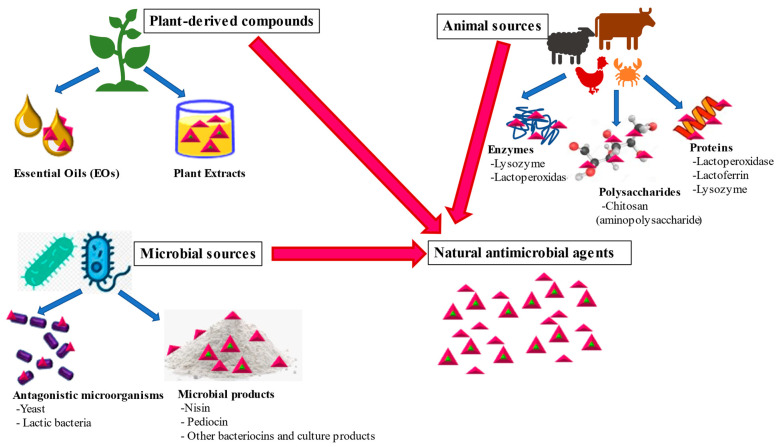
Sources of natural antimicrobial agents as additives in edible packaging systems.

NAMAs are in vogue in the present-day scenario, owing to their potency and safety, which is in harmony with the current needs of the food packaging industry, which is looking for sustainable alternatives. Nevertheless, their abrogating effect on the aroma of food being incorporated together with their depletion in functionality due to early infusibility makes their practical usage in the food industry constrained. In this context, the natural antimicrobial additives obtained from plants, animals and microorganisms are attracting the attention of scientists (Figure 1). These derived compounds have wide application to fresh and processed food products to hamper deterioration, amplify storage life and assure the safety of the products [12].

To overcome this constraint, edible films are supplemented with these natural additives. In recent years, encapsulation of these bioactive by various techniques such as nano-encapsulation, spray drying, polymerization, etc., have been identified as interesting strategies for improving the performance of edible films [13]. The potential application of edible packaging for the embracement of NAMAs and improved controlled release of these additives in the food system is highlighted under this heading.

Incorporating synthetic antimicrobials in edible films is a hot debatable topic because of their potential adverse effects. Hence, the consumers are more intended to go for natural and harmless preservatives derived from various natural sources like bacteriocins, enzymes, organic acids, essential oils and so on.

### 2.1. Plant-Derived

Plant extracts and essential oils are in use from ancient times to improve taste and create distinctive flavors and augment the storage life of food products by averting the proliferation of pathogenic and spoilage microbiota and oxidation of food components [14,15]. A higher amount of secondary metabolites such as aldehydes, ketones, phenolic compounds, acids, highly lipophilic and volatile nature have enhanced their potency. Due to the versatile content of NAMAs, they have inherent potential as natural agents for food preservation. Essential oils derived from various spices have been categorized as Generally Recognised as Safe (GRAS).

In spite of all these advantages, direct incorporation of essential oil in food products is restricted because of their highly volatile nature, hydrophobicity and loss of flavour, susceptibility to oxidation and photo-thermal degradation [16]. Therefore, to circumvent these limitations, it is contemplated to summate these in the packaging material like edible films. The use of edible films having a blend of essential oils or plant extracts is gaining popularity. Essential oils entrapped in edible films help sustain release and curb their volatility, thereby remodeling their efficacy. For the controlled release of antimicrobials and to minimize loss during film manufacturing and storage, the usage of hydrocolloids like chia seed mucilage with cyclodextrin is advocated [17]. When embodied with Ginkgo biloba extract, Gelatin edible films were effective against *Staphylococcus aureus* and *Candida albicans* [16]. A remarkable antibacterial effect was observed against *Pseudomonas* spp., mesophilic and psychrophilic bacteria due to the incorporation of grape seed extract and carvacrol microcapsules in chitosan films [18]. Millet starch edible films with polyphenolic clove essential oil offered a novel strategy to boost antioxidant and microbiological protection, extending food shelf life. These films showed substantial antimicrobial activity against *Escherichia coli, Pseudomonas aeruginosa, Enterobacter* sp., *Bacillus cereus, Staphylococcus aureus* and *Trichoderma* fungi. The findings revealed that starch-based edible films containing varying concentrations of clove essential oil exhibited inhibitory efficacy against both gram-positive and gram-negative bacteria. The inhibitory effect increased proportionally with the amount of clove essential oil [19]. In addition, the edible films also help in improving their contact area with food. Several researchers have reported adopting critical oils and plant extracts as NAMAs in edible packaging for several food products (Table 1).

### 2.2. Animal-Derived

The literature has deduced that there are a number of polymeric compounds obtained from animals that exhibit inherent antimicrobial activity, such as whey proteins, protein hydrolysates, chitosan, bioactive peptides, etc. Chitosan has displayed antagonistic behavior against bacteria such as *Bacillus cereus, Salmonella typhimurium, Listeria monocytogenes, Escherichia coli, Staphylococcus aureus* and various mold and yeast such as *Botrytis cinerea, Candida lambica, Fusarium oxysporum*, *Rhizoctonia solani* [20]. Chitosan’s antimicrobial effect can be attributed to the interaction of its amino groups (positively charged) and the cell membranes of microbiota. Consequently leading to the development of perforations in the cell membrane, thereby causing leakage of the intracellular substances. Additionally, chitosan also exhibits remarkable film-forming characteristics attributed to its polycationic nature. Furthermore, a striking observation was made by Nouri et al., 2018 [21] that chitosan can chelate metals in the proximity of bacteria, preventing critical nutrients from moving, hence inhibiting the growth of bacteria. Other factors affecting the action of chitosan are pH, target microorganism, time of exposure, degree of acetylation and cationic nature [22].

Whey protein is obtained as a by-product of the dairy processing industry and showcase high biological activities. Whey is a dairy industry by-product with strong biological activity. Bioactive peptides derived from whey also illustrate antimicrobial action, but their exact mechanism of action is still undiscovered. Like chitosan, its activity confides in pH, the temperature of treatments and the presence of fat [23]. In a research experiment by Liu et al., 2019 [24], whey protein nanofibrils with titanium dioxide nanotubes were added in edible films to study their antioxidant and antimicrobial effect on the storage life of chilled meat. It was deduced from the results that the diameter of inhibition zones for *Listeria monocytogenes, Staphylococcus aureus, Escherichia coli* and *Salmonella enteritidis* was ≥10mm, thereby contributing to the increased storage life of chilled meat [25].

Lactoperoxidase is a protein (glycoprotein enzyme) present in raw milk, colostrum, saliva and other secretions. Owing to its bactericidal or bacteriostatic qualities, lactoperoxidase (LPO), one of the most important enzymes employed as a natural antimicrobial agent, has piqued attention in food packaging. When trout fillets were coated with lactoperoxidase system incorporated into chitosan solution, the number of *Shewanella putrefaciens, Pseudomonas fluorescens* and psychrotrophic and mesophilic bacteria were considerably reduced. Hence, increasing the shelf life of fillets by at least four days [26]. Lactoferrin is an iron-binding glycoprotein classified as a member of the transferrin family and is present in several mammals’ milk and other fluids. It showcases both bactericidal as well as bacteriostatic activity against a wide range of bacteria. Its iron binding capacity deprives certain bacteria like *Salmonella* spp, *Listeria monocytogenes, Bacillus stearothermophilus, Escherichia coli, Bacillus subtilis* and *Shigella dysenteriae* of the nutrients they need to flourish. When applied on sausage, bacterial cellulose filme with adsorbed lactoferrin depicted an excellent bactericidal efficiency against *Escherichia coli* and Staphylococcus aureus [27]. Similarly, an enzyme lysozyme in egg white, blood and milk possess anti-activity against Gram-positive and gram-negative bacteria. Biocomposite edible films made up of hydroxypropyl methylcellulose, with different concentrations of chitosan and bioactive cystatin/lysozyme preparation, showcased antagonistic action against *Micrococcus flavus*, *Bacillus cereus*, *Escherichia coli*, *Pseudomonas fluorescens* and *Candida famata* [28].

Edible packaging components with bioactive peptides and protein hydrolysates have exhibited accomplished efficacy against the multiplication of spoilage/pathogenic microflora and lipid oxidation, thus promoting the concept of safe food.

### 2.3. Micro-Organism Derived

Bacteriocins, low molecular weight peptides, are recognized as natural antimicrobial compounds known for their effectiveness against microorganisms. These are a wide group of antibacterial chemicals produced by Lactic acid bacteria and bacteria representing genus *Bacillus* [29]. A number of bacteriocins such as nisin, lacticin, pediocin can be imbibed into the edible packaging to suppress the multiplication of pathogens and spoilage microbes. Edible films with bacteriocins reduce spoilage bacteria’s development on food surfaces by assuring direct contact of packaging with the food product. Sustained release of bacteriocins from edible packaging onto the surface of food is an excellent technique compared to the dipping or spraying of bacteriocins [30].

Due to its broad spectrum of activity, nontoxic and nonallergic nature, nisin is of the greatest utility in advancing antimicrobial packaging. It is produced by Lactococcuslactis and is grouped under the category GRAS. To restrain the growth of *Vibrioparahaemolyticus* ATCC 17802 and *SalmonellaTyphimurium* ATCC pathogens on sea food, a research study was planned by Pattanayaiying et al., 2019 [31]. A gelatin-based edible film containing nisin Z was applied on tiger prawn and bigeye snapper. It was observed that films containing nisin Z were effective against these pathogens in chilled and frozen sea food even after 21 days of storage. Cellulose-based edible films with bifidocin A acted as a promising method to suppress the enumeration of Pseudomonas and Enterobacteriaceae and enhance the storage life of fresh Spanish mackerel fillets to 12 days without getting deteriorated [24].

Bacteriophages have a multifaceted application in the food industry. They have emerged as a viable option for pathogen eradication from food sources. To be successful, phages must exhibit stability when exposed to experimental conditions [32]. Firstly, a phage must be incorporated in a fashion that enables them to access the target pathogens. Encapsulation of bacteriophages in edible films is emerging as a promising approach to limit the growth of pathogenic or spoilage bacteria (Table 1). Bactericidal and inhibitory potential of Listex P100 was investigated against various concentrations of *Listeria monocytogenes* in three distinct matrices namely sodium caseinate, sodium alginate mixed with gelatin and polyvinyl alcohol. It was demonstrated that bacteriophage had a strong antibacterial potential against *L. monocytogenes* with 4.40 and 6.19 log reduction values. To extend bacteriophages to packaging systems, current research has investigated means of maintaining phage stability and activity [33]. Kalkan [34] tested the effectiveness of encapsulated bacteriophages in methylcellulose edible films against *Vibrioparahaemolyticus* ATCC 17802 in raw fish fillets. The researcher achieved good results against the specific pathogen and was also successful in maintaining the stability of bacteriophage in the film. In another thought-provoking recent study, *Salmonella enteritidis bacteriophage* Felix O1 was assimilated in polyvinyl alcohol coatings and was found to maintain antagonistic activity (10^6^ titer) against *Salmonella* bacteria [35]. Hence, these revolutionary, biodegradable and novel edible packagings with phages can be a viable alternative for limiting the growth of food pathogens.

Lactic acid bacteria are known to exhibit antifungal activity because of the production of certain antimicrobial metabolites in fruits and vegetables. In one experiment, *Lactobacillus plantarum* was tested for its ability to prevent fungal deterioration on grapes in conjunction with edible coatings. Various combinations with or without *L. plantarum* were examined, with the primary components of the coating matrices being pregelatinized potato starch or sodium caseinate. It was observed that *L. plantarum* in potato starch formulation exerts antifungal activity against *Botrytis cinerea* [36].

The quality and nutritional parameters of ready-to-eat strawberries stored at 4 °C for 10 days were studied by applying chitosan or chitosan and Palmaria palmata Kuntze algae-based edible films. The results illustrated that chitosan-based edible films were able to reduce microbial load and nutritional losses. Moreover, the addition of *P. palmate* enhanced the ascorbic acid and anthocyanin content considerably [37]. In a study by Luo et al., 2020 [38], strawberries kept in cold storage were coated by *Laminaria japonica,* a brown seaweed. It was displayed that the treatment helped curb the degradation of ascorbic acid and polyphenols, hence controlling antioxidant capacity.

**Table 1:** Overview of NAMAs derived from various sources in edible packaging and their effects.

**NAMAs**	**Targeted Micro-Organisms**	**Edible Film Matrix**	**Food Product**	**Findings**	**References**
**Plant-Derived Antimicrobials**					
Rosemary essential oil	Coliform bacteria	Whey protein concentrate	Fresh spinach	Reduced the total microbial and coliform count to 0.57, 0.23 log CFU/g, respectively. 2. Loss of chlorophyll also decreased.	[14,15,39]
Nanoemulsion of essential oil from cumin	*Listeria monocytogenes; Escherichia coli; Salmonella typhimurium*	Chitosan film	Refrigerated beef loins	1. Enhanced the storage life of beef loins by inhibiting the proliferation of mesophilic, psychrophilic, *Enterobacteriaceae* and lactic acid bacteria. 2. Augmented the antioxidant activity.	[40]
Clove essential oil and kojic acid	Aerobic bacteria	Fully deacetylated chitosan edible films	White prawn shrimp kept in cold storage	1. Dramatically reduced the proliferation of aerobic bacteria 2. Melanosis and color changes were also deaccelerated.	[41]
Chitin nano fiber and Ajowan (Trachyspermumammi) essential oil	*Pseudomonas; Staphylococcus aureus*, lactic acid bacteria, yeast and molds	Gelatin and carboxymethyl cellulose films	Raw beef held at refrigerated conditions	1. Discouraged the multiplication of pathogenic microorganisms. 2. In addition, also had a complimentary effect on sensory properties.	[42]
Garlic essential oil. Oregano and garlic essential oils	Spoilage bacteria *Escherichia* *Coli; Salmonella enteritidis;* *Listeria monocytogenes;* *Staphylococcus aureus*; *Penicillium* spp	Chitosan and whey protein amalgamated films Whey protein isolate	Vacuum-packed sausages Kasar cheese slices	1. Retarded the growth of bacterias responsible for spoilage. 2. Decrement of fat oxidation Exhibited an antimicrobic action against the majority of pathogenic bacteria	[43,44]
Oregano essential oil and resveratrol nanoemulsion		Pectin edible coating	fresh pork loins	1. Prohibited growth of microorganisms. 2. Enhanced storage life 3. Retainment of sensory characteristics of pork loins.	[45]
Musk lime extract	*Pseudomonas aeruginosa*; *Vibrio parahaemolyticus*	Chitosan film	Squids	Expanded the inhibition zone of these gram-negative bacteria	[46]
Ginger essential oil	Aerobic Psychrophilic bacteria	Sodium caseinate coating	Chicken breast fillet	Antibacterial activity was significantly elevated at *p* < 0.05.	[47]
Cinnamon essential oil	*Staphylococcus aureus; Escherichia coli*	Sodium alginate and carboxymethyl cellulose films	Banana	Showcased exceptional antimicrobic activity.	[48]
Pomegranate peel extract	*Staphylococcus aureus; Salmonella*	Starch base films	NA	Appreciably constrained the growth of both bacteria	[49]
Herba Lophatheri extract	*Staphylococcus aureus; Escherichia coli*	Chitosan films	NA	Inhibitory zone diameter increased by 17.02 and 19.28 percent against *Staphylococcus aureus* and *Escherichia coli*, respectively	[50]
Thymol	*Botrytis cinerea*	Chitosan coating	Cherry tomatoes	Coating of cherry tomatoes by quinoa protein/chitosan) showcased antifungal action against *Botrytis cinerea* after seven days of storage at 5 °C (*p* < 0.05)	[51]
Turmeric extract	*Salmonella; Staphylococcus aureus*	Chitosan films	NA	Improved activity against these bacteria after the impregnation with turmeric extract	[52]
Propolis extract	Total viable count; Psychrotrophic bacteria; *Pseudomonas* spp.; Lactic acid bacteria; *Enterobacteriaceae*	Chitosan films with cellulose nanoparticles	Minced beef	Caused delay in microbial growth	[53]
Carvacrol essential oil	*Escherichiacoli*	Thermoplastic starch films	NA	Substantial and noticeable antimicrobial activity against *E.coli* because of loss of homeostasis and fractional dissolution of the cell membrane.	[54]
Cinnamon essential oil and nano titanium dioxide	*Escherichia coli; Salmonella typhimurium; Staphylococcus aureus*	Sago starch films	Fresh pistachio packaging	Outstanding competence to stop the multiplication of food spoiling bacteria Improved mechanical characteristics of films	[55]
Nanoemulsions of polyphenols (curcumin, gallic acid and quercetin)	*Escherichia coli; Salmonella typhimurium*	Gelatin and carrageenan composite film	Chicken meat	Increased storage life of broiler meat up to 17 days by inhibiting the growth of pathogens.	[56]
**Animal-Derived Antimicrobials**					
Casein phosphopeptides	*Staphylococcus aureus; Bacillus cereus*	Gelatin based films	NA	1. Exhibited significant inhibitory effect against these gram-positive pathogens. 2. Represented increases antioxidant activity	[57]
Gelatin and cinnamon essential oil	*Escherichia coli; Staphylococcus aureus*	Chitosan-based films	NA	1. Showed excellent mechanical and antibacterial characteristics 2. Rate of antimicrobial activity was 98%	[58]
Fish protein hydrolysates Fish protein hydrolysates and clove essential oil	Mesophillic; psychrophilic; coliform bacteria; yeast; mold *Staphylococcus aureus; Yersinia enterocolitica; Aeromonas hydrophila; Debaryomyceshansenii; Listeria innocua.*	Protein-based edible coatings Agar based films	Chilled Bonito Fillets Flounder fillets	Imparted excellent inhibition to the growth of mesophilic, psychrophilic, coliform bacteria as well as yeast and mold 1. Enhanced shelf life 2. Effective against the listed microorganisms	[59,60]
Activated Lysozyme	*Listeria innocua*	Whey protein and oleic acid films	Smoked salmon slices	Decreases bacterial load and increased shelf life even after opening packet at refrigerated temperature	[61]
**Microorganism Based Antimicrobials**					
Natamycin and *Pitanga* leaf extracts	*Aspergillusflavus*; *Aspergillus parasiticus*	Cassava starch and chitosan	Casting method	1. Imbibition of *Pitanga* extract did not affect the mechanical properties of films, but the addition of natamycin decreased flexibility. 2. Uptrend in antioxidant activity 3. Significant improvement in antifungal properties	[62]
Nisin Nisin and clove essential oil	*Staphylococcus aureus;* methicillin-resistant *Staphylococcus aureus* (MRSA) strains *Pseudomonas* spp.	Carrageenan and chitosan Chitosan	NA Pork patties	Bactericidal efficacy was 90% and 99% of planktonic and biofilm cells, respectively, against these two strains of bacteria 1. Strong antimicrobial action against *Pseudomonas* sp. was reported. 2. Shelf life was enhanced by two times 3. Synergistic effect on antioxidant activity also	[63,64]
*Lactococcus lactis* Cell-free supernatant of *Lactococcus lactis*	*Staphylococcus aureus* *Staphylococcus aureus* ATCC 6538; *Escherichiacoli* ATCC 2592	Sodium alginate/ sodium carboxymethylcellulose films Sodium alginate or sodium carboxymethylcellulose film	Tryptone soya agar NA	1. Repressed the growth of *Staphylococcus aureus* for seven days at 4 °C. 2. Reduction in transparency and gloss Robust antagonistic effect against *Staphylococcus aureus* and *Escherichia coli*	[65,66]
Cocktail of six lytic bacteriophages	*Escherichia coli*	Whey protein concentrate film	Meat	1. *E.coli* was reduced to an undetectable level. 2. Bacteriophages were highly stable in whey protein concentrate films	[67,68]
Bacteriophage vB_EcoMH2W	*Enterobacteriaceae* family	Chitosan-based coating	Tomatoes	Significant reduction in the growth of bacteria by 3 log cycles when stored for one week.	

## 3. Film/Coating Formation Methods

### 3.1. Casting Method

**Figure 2:**
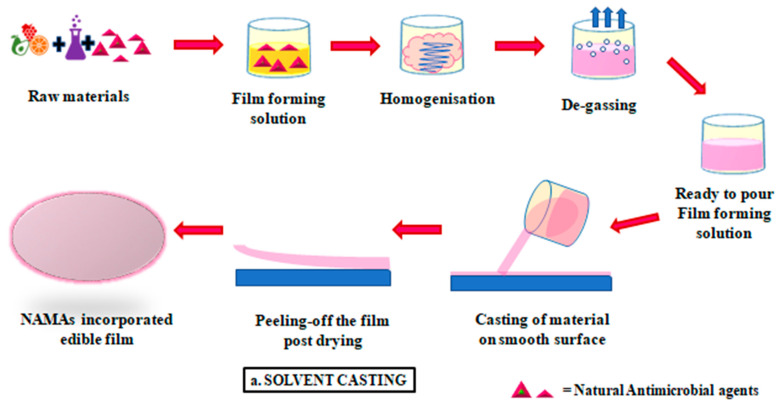

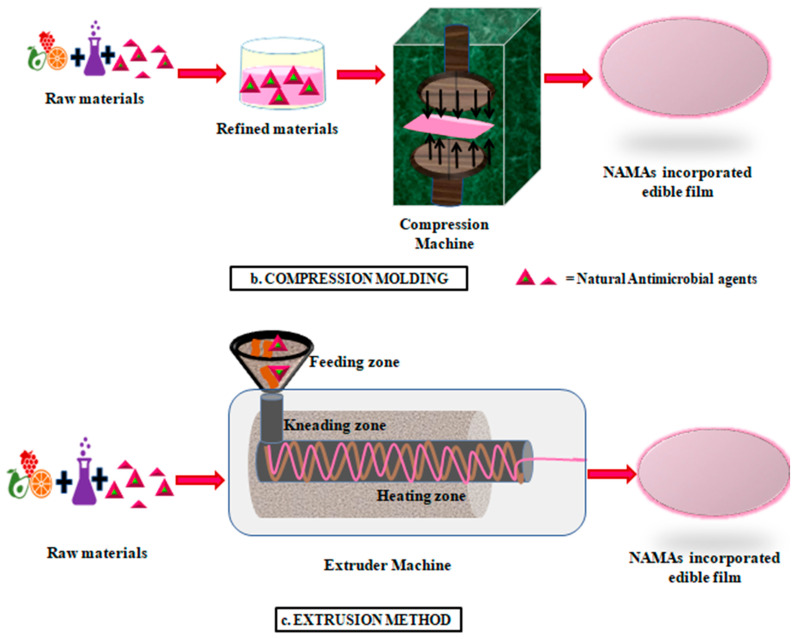
Various methods of film formation (**a**) solvent casting (**b**) compression moulding (**c**) extrusion method.

A lab or pilot scale method, also known as solvent casting, is a rather simple and one of the most common techniques of edible film formation. This method has been adapted at an industrial scale with various animal and plant-based materials as the main source of biopolymers [69]. Starch, cellulose, pectin, gums, chitosan, agar, alginate, dextran, gelatin, casein, whey protein, waxes, paraffin and glycerides are the common biopolymers used in the manufacture of edible films by solvent casting, along with plasticizers such as glycerol, aloe, resins; and chaotropic agents like urea [70]. After selection of suitable ingredients, the biopolymer is solubilized in a suitable solvent along with the NAMAs, followed by casting of solution the mold. Post this step is followed by degassing and drying is completed before the film is, finally, peeled off from the surface [71], as depicted in Figure 2a. This method has the advantage of being simple, easy in operation, low cost and environment friendly [72]. The outcome of the casting process is a function of various factors like atmospheric conditions, equipment, time and temperature combination used [73].

### 3.2. Compression Molding

Either thermo-compression or ultrasonic compression binds the film-forming materials into a desirable shape and thickness [74]. An ultrasonic welder is used for welding the film materials, which have been previously refined, as depicted in Figure 2b. Post compression, the welded materials are cut and processed to elaborate sustainable edible packaging systems [58]. Various raw materials to form nanocomposites can be used as mentioned in Section 3.1, but starches, particularly, cassava starch have excellent properties and show less degenerative changes when subjected to compression molding [75]. This technique has not yet gained popularity for manufacturing edible films but is a fast and economical method and needs to be adapted to suit the edible film packaging industry [71].

### 3.3. Extrusion Methods

To improve the mechanical and water vapor barrier properties of the polysaccharide-based films, lipids are added to enhance the hydrophobicity of the films [76]. Co-extrusion blowing is a suitable technique to attain the desired results when multiple sources of biopolymers, most commonly lipids and starches, are being used along with other additives [77]. In comparison to solvent casting and compression methods, extrusion is a further more active technique. The process can be broken down into three main steps: feeding, kneading and heating, as depicted in Figure 2c. Firstly, the film-forming material and NAMAs are brought to the feeding zone and degassing is done by using compressive force. Secondly, the materials are further compressed by increasing the pressure and temperature to attain specific physical attributes. Lastly, heating occurs in the final section of the extruder, where parameters like temperature, shear rate and pressure are highest. Hence, it can be rightly said that the physicochemical characteristics of the film so formed are an outcome of the events of the heating section. Parameters such as configuration of the screw, the ratio of screw diameter and length, rate of feeding, moisture percent, speed of the screw, etc., can be tailored by the end use of the edible film so formed [78].

## 4. Film/Coating Application Methods

### 4.1. Knife Coating

**Figure 3:**
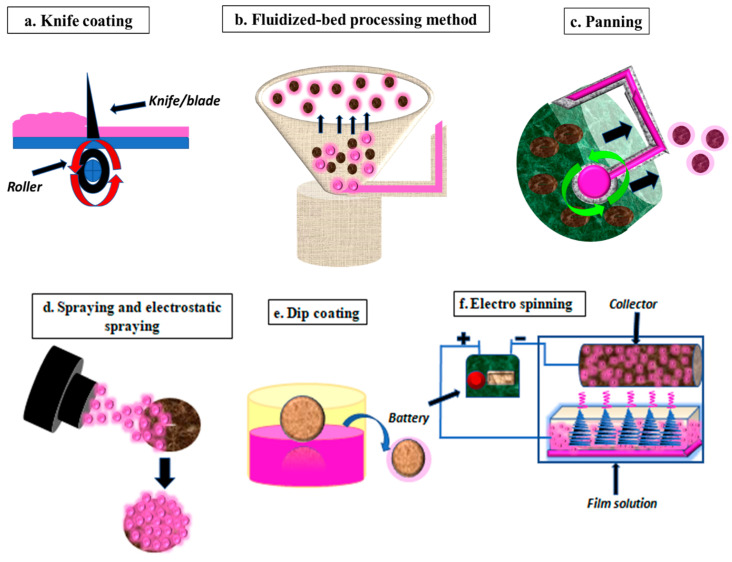
Various methods of film application (**a**) knife coating (**b**) fluidized-bed processing method (**c**) panning (**d**) spraying and electrostatic spraying (**e**) dip coating (**f**) electro spinning.

This method of film application has been adapted on a commercial scale. It comprises a fixed knife that carefully deposits a layer of film-forming material over the smooth casting surface or food product positioned beneath the knife, as depicted in Figure 3a. The barrier properties of the film so formed are chiefly determined by the wet film characteristics. The surplus wet film material is held back in the pool situated behind the knife. The width of the film can be adjusted by fine controls that affect the height of the knife above the smooth casting surface or food product. This technique for coating a specific substrate/food product is optimized after taking into consideration parameters such as viscosity, solid content, moisture, the thickness of wet formulation, ingredients, temperature, NAMAs and to be coated, into consideration [79].

### 4.2. Fluidized-Bed Processing Method

This process can be divided into three categories: top spray, fluidized bed and bottom spray. This method is used to coat materials having extremely low density or exceptionally small sizes. The modus operandi involves feeding the small particles to be coated in a rotating fluidized bed. A centrifugal force shoves the particles post-coating to the wall of the equipment. There is a provision for the airflow into the instrument. Overall, airflow and centrifugal forces counter the total force upon the food particles, as depicted in Figure 3b. The advantage of this technique involves the freedom to choose between a wide array of coating substances and the number of layers to be deposited. This technique is yet to gain popularity due to the complexity and cost of the technology involved [71].

### 4.3. Panning

The given technique makes use of a rotating pan containing the food product to be coated. The solution to be coated is then poured upon the pan and the food product is toppled to coat the desired number of layers over it, as depicted in Figure 3c. This is followed by drying the coated product airflow or thermal treatment. The method was commonly developed for drugs and is still being optimized for use in the food packaging industry [71].

### 4.4. Spraying and Electrostatic Spraying

As the name suggests, this conventional technique involves spraying low viscosity film-forming solutions upon the food products, as depicted in Figure 3d. Spraying is suitable for large surfaces and is usually done under high pressure via a spray gun to the tune of 60–80 psi. A multilayer coat of edible packaging can be achieved via this technique [80]. In addition to other parameters, the size of the coating droplet also depends upon the nozzle and spray gun, air flow rate, rate of flow of liquid, temperature and humidity of inward air [81].

An upgrade to the classic spraying is the use of electrostatic force for the application of edible coatings. Electrostatic spraying ensures uniform and fine particle size of the film-forming material, less than 100 nm. Spraying remains the most efficient method to ensure desired layers, proper film formulation utilization and uniformity. Drying time, temperature and method are key components guiding the outcome of spray drying [82].

### 4.5. Dip Coating

This is the method of choice for coating irregular food surfaces. This method is highly acceptable in the food packaging industry because of its ease of operation and cost economics. The coating is achieved by plunging the substrate in the film-forming solution, as depicted in Figure 3e. Surfeit coating is removed either by draining or by deploying a dryer to attain the coated end product. The entire procedure is quick and lasts for 5–30 s. Amongst all the methods of application of edible coatings, dipping ensures high thickness coatings that function of surface tension, density and viscosity of the film-forming material [83].

### 4.6. Electro Spinning

Electro spinning is an advantageous technique of application of edible coatings by providing better control over film structure, high ratio of surface to volume, lower price and improved porosity. This method utilizes a high voltage electric field to bring together the film-forming material upon the desired substrate, as depicted in Figure 3f. It is highly recommended to develop encapsulated edible films containing NAMAs that are sensitive to high temperatures. The only disadvantage is the low productivity rate, which discourages its commercial application [84].

## 5. Application of NAMAs Films in the Food Industry

Edible, including organoleptic characteristics. Abundant literature is available regarding the applicability of these antimicrobial films in a variety of food systems. Therefore, this focus is given on compilation of the latest advancements in the relevant field (Table 2).

## 6. Effect of NAMAs on Physico-Chemical Properties of Edible Films

### 6.1. Barrier Properties

Incorporation of NAMAs may or may not enhance the mechanical properties of edible films. It has been frequently reported that the mechanical properties of the antimicrobial film are strongly dependent on the concentration of antimicrobial compounds. Higher concentrations of NAMAs are expected to lead to lower film strength and greater film extensibility because high amounts of NAMAs inclusion may help to enhance the plasticizing effect of the edible film, consequently improving the film’s extensibility.

However, some researchers have reported that the inclusion of antimicrobial agents above a certain limit led to a decrease in the film stretch [109,110]. Thus, before making an antimicrobial film, the concentration of antimicrobial agents should be considered. Good mechanical properties are among the basic requirements for the antimicrobial film as active food packaging since poor extensibility or strength may lead to premature failure or cracking during production, handling, storage or use. It has been reported that antimicrobial agents could play a role as a plasticizing agent in the films, leading to the interaction between intermolecular reduction and the enhancement of macromolecules’ mobility [111]. Recently, several researchers have reported that the addition of antimicrobial compounds could influence the optical properties of an edible film by decreasing lightness and film transparency values and increasing redness and yellowness values correlated with the increasing content of antimicrobial agents [18,112].

Tensile strength tends to increase until white turmeric extract is 7% and drops sharply on the addition of white turmeric extract 11% and is inversely proportional to the decreasing elongation value. The sharp decrease in tensile strength of edible white turmeric extract film 11% is influenced by the thickness, which makes the film stiffer than the others so that when pulled; it is not elastic and easily broken. The addition of white turmeric extract causes edible film thickness to increase because the addition of white turmeric extract will increase the total amount of solids [113]. The addition of white turmeric extract to edible film reduces elasticity and is easily tearable.

Cardoso et al. (2017) [114] prepared poly butylene adipate co-terephthalate edible films with essential oregano oil as antimicrobial agent and extrusion and evaluated morphological, physical, mechanical, water barrier, thermal and structural properties of films. It was reported that the essential oil content improved the water vapor permeability while no noticeable change was observed in the mechanical and thermal properties of the films. The protein hydrolysate and clove essential oil were added to agar film applied to flounder fillets. The addition of protein hydrolysate improved different barrier properties, viz., water vapor permeability, water solubility and elongation at break, whereas clove oil films had reduced the transparency [47]. Sweet potato starch-based edible coatings with varying concentrations (2–6%) of thyme essential oils significantly improved different properties, including higher L value, hardness, cohesiveness, gumminess, springiness and chewiness and resilience values throughout the storage time than the uncoated samples [88]. Akin to these findings, the physicochemical properties of chitosan films loaded with essential oregano oil were reported to have improved thickness, higher elasticity, reduced puncture and tensile strength and lower moisture barrier properties than pure chitosan films [115]. The addition of tarragon essential oil in whey protein isolate (WPI) based edible films improved moisture barrier properties, water-solubility, color and transparency of the film; decreased puncture resistance and puncture deformation [116]. The incorporation of gelatin films with ginko-biloba extract increased the UV-visible shielding performance of films and enhanced mechanical properties. The presence of ginko biloba extract as an antimicrobial agent in edible films resulted in cross-linking between the amino group of the protein and the phenolic group in the extract that caused an increment in rigidity and tensile strength of the film decrement in elongation at break. Due to the hydrophobic nature of ginko biloba, the moisture permeation properties of the films were negatively affected [18].

Similarly, Yao et al. [117] also noticed decreased WVP in fish gelatin–chitosan films incorporated with D-limonene because of its hydrophobicity that possibly hindered water transfer through the films. From the best formulation of edible film-making, the antimicrobial compounds were added. There were two antimicrobial compounds, garlic extract and chitosan and three levels of chitosan concentration (0; 15; and 30%) and garlic extract (0; 0.2; and 0.4%). Each antimicrobial activity was carried out by in vitro assay using the disk diffusion method (diameter 5 mm). The best edible film resulted from 4% starch acetate with high tensile strength (1.635 MPa) and elongation (49.101%) values [118].

### 6.2. Bio-Degradability

Films and coatings prepared from biodegradable materials are increasingly being used in the food packaging industry in response to the growing demand for sustainability and ecological safety. Biodegradation kinetics depends on the polymer used (molecular weight, structure) and additives used (plasticizers, fillers). An edible film incorporated with NAMAs is utilized to control microbial growth in foods, leading to shelf-life extension and improved microbiological safety of food products. The concept of antimicrobial packaging systems is considered with a focused development on biodegradable films, mainly polysaccharides and protein-based materials. Among various approaches for biopolymer improvement, the use of nanotechnological concepts is one of the important development [119,120]. As a result, nanotechnology-based food packaging solutions have an environmental benefit over traditional equivalents that use plastic barriers, while functional components like antibacterial agents increase product shelf life. It also helps identify different spoilage indications, such as off-flavors, color and hazardous food contaminants [121]. The bio-nanocomposite materials are biocompatible, biodegradable and have superior mechanical and physicochemical properties thus, serving better replacement to the traditional plastics as packaging materials.

In addition to this, biopolymers such as chitosan, carboxymethylcellulose, starch, alginate, casein, carrageenan exist as suitable candidates for solving the ecological problem of plastic packages their superior biodegradability and non-toxic characteristics. However, these have some drawbacks that without the addition of suitable additives, the mechanical properties of edible films are inferior, viz., poor moisture barrier properties. Consequently, embedding different bioactive/functional ingredients as additives improves thermal and mechanical properties with enhanced biodegradability characteristics of the biopolymer. Among these biopolymers, chitosan naturally having antimicrobial properties, but cellulose lacks antibacterial activity and, therefore, it must be incorporated with antimicrobial agents. These antimicrobial agents behave as reinforcing components, improving mechanical properties [15,122,123].

Polylactic acid (PLA) is biosourced and biodegradable and can be used as the base for antimicrobial coated films [4,124]. Among the biodegradable polymers, poly(lactic acid) (PLA) has received great attraction as it can be easily degraded by hydrolysis and enzymatic reactions without leaving harmful or toxic residuals.The Food and Drug Administration has also approved this biopolymer as safe, being produced from renewable products such as corn and sugarcane. It bears superior mechanical properties similar to PS and PET with an additional advantage of biodegradability [125].

### 6.3. Edibility

The customer acceptability of edible films depends not only on the functional properties but also on organoleptic characteristics and the edibility of film. Edible films formulations should not negatively impact the organoleptic and nutritional properties in the food packed. The films should be transparent, colorless, odorless, tasteless and glossy [126]. However, much attention is required to improve the organoleptic properties and, hence, its edibility. Lack of awareness and misconception about ingredients of edible films can reduce consumer acceptance. Vegetarians may have an aversion towards animal-derived edible films.

Furthermore, another important aspect that should be considered is the toxicity and allergenicity caused by different types of ingredients of edible films. Essential oils, frequently employed in edible coatings as antibacterial agents, are categorized and registered as GRAS by the European Commission and the United States, may cause allergic reactions and oral toxicity [127]. Therefore, it is necessary to balance the effectiveness of essential oil or plant extract dose and the risk of their toxicity [121,125]. Overall, since edible films and coatings become part of the food to be consumed, all materials used in these products must be appropriately declared on the label [128].

Although the toxicity of natural antibacterial agents is low, the production cost is high, hindering their wide applications. However, the toxicity of synthetic agents should not be ignored. Reducing the toxicity of synthetic material is also an important subject in need of further research. With the development of antimicrobial packaging materials, highly comprehensive, systematic and unified standards are needed to evaluate their antimicrobial activity and food safety. Antimicrobial packaging materials should also be provided with smart technology, such as indicators, to show the degree of bacterial infection in food or its micro environmental factors, such as temperature, pH value and humidity and to meet high requirements and enrich food packaging applications [103].

However, several research studies also report that incorporating NAMAs in edible films not only improves the functional and mechanical properties of films, but also the edibility characteristics. A chitosan-based edible film containing grape seed extract enhanced the sensory qualities of fresh chicken flesh [129]. Fermented soybean paste was used as a flavor coating in pastirma efficiently reduced the formation of sulfur or sotolone compounds that cause unpleasant odor [130]. In fish, preserving sensorial quality was also reported after wrapping in chitosan-based coating incorporated with a lactoperoxidase system [26]. Another relevant outcome obtained from active coating containing resveratrol was reported to have less intense fishy off-flavor preserve the coated sea bass fillets for four weeks [131].

Lack of evidence on edibility and biodegradability, organoleptic aspects, insufficient legal aspects, fear of toxicological and health effects, inadequate marketing, public awareness, cultural issues, etc., can affect the consumer acceptance of edible films. Future research on edible films should also consider these aspects to improve commercialization success.

### 6.4. Biocompatibility

The incorporation of functional or other bioactive ingredients in the biopolymers (chitosan, alginate, whey protein, etc.) matrix not only strengthens the film, but these functional agents act synergistically with matrix ingredients to enhance the functional properties of edible films viz, moisture barrier properties, enhanced antioxidant potential and antimicrobial properties. Therefore, it becomes necessary to analyze the compatibility between antimicrobial agents or other functional ingredients with the biopolymer matrix to prepare a successful edible film/coating. Compatibilization between antimicrobial agents/functional additives and the polymeric matrix is the biggest challenge for developing antimicrobial packaging [132].

About several advantages, these different NAMAs, essential oils and polyphenolic compounds are known to have a major drawback of migration into packed/wrapped food products thus, causing undesirable alterations in sensory characteristics, particularly undesirable alterations in flavor and aroma due to mixing of volatile and non-volatile components, hence restricting their applicability in different foods. However, the emergence of micro- and nano-encapsulation techniques in the arena of edible films/coatings serve as a very useful tool to enhance the compatibility of different functional additives and further widen the applicability of such edible films/coatings. Therefore, it is the need of the hour that awareness among the consumers of these types of edible coatings/films needs to be increased to broaden and diversify their applicability in food preservation [94].

Edible films containing NAMAs may increase or decrease the organoleptic acceptance of the product. Astringency was noticed in edible films containing high amounts of apple peel polyphenols, reducing the palatability of films [133]. On the other hand, coatings may improve the appearance by imparting glossy food surfaces, improving the aesthetic quality of food [134].

The variation of glass transition temperature (Tg) is one of the effective indicators of the compatibility of polymers in edible films. The presence of only one Tg (glass transition) could indicate that the sago starch and gelatin films are compatible [135]. These phenomena with only one Tg observed in starch caseinate blends plasticized with glycerol or sorbitol was also reported by Arvanitoyannis and Biliaderis (1998) [136] and Su et al. (2010) [137], stating that presence of one glass transition for different polymers of a film in DSC scan indicates good compatibility among component polymers. However, still, more research is required to study the compatibility between various bioactive additives and biopolymers.

## 7. Potential Advantages and Pitfalls of Antimicrobial Edible Films/Coatings

With the advent of edible antimicrobial films, a growing concern is the extrapolation of results obtained in the lab experiments and their applicability in real situations. Generally, the experiments conducted in the laboratory are done by using food simulants that have a lesser degree of complexity than existing food systems. Different food constituents like fat, minerals, vitamins, salt and proteins may interact with antimicrobials and modify their mode of action. In addition, the storage conditions, temperature, relative humidity in actual also have a significant impact on their sustainable release and properties of NAMAs from edible films. Moreover, assessment of edible antimicrobial packaging from safety point of view is very important to complement food safety with the prevention of the development of resistant strains of microorganisms. Table 3 encompasses the potential advantages and pitfalls of NAMAs in edible packaging.

## 8. Public Demand, Future Prospects

It is a well-established fact that the consumer demand for whole, safe, minimally processed, sustainable and healthy foods are rising. The concept of food safety has been around for a long, but the safety of food packages that pose serious health risks to consumers is being recognized [6]. The conversant consumers today raise concerns about the very nature of food products and the package that directly comes in contact with the food. Hence, it becomes a priority to ensure the same. NAMAs can be extensively used in biopolymer-based films to meet consumer demand for food safety. Additionally, these agents in films can mask the off-flavors and improve the sensory characteristics, preventing browning and pathogenic changes in the food products they contain [138].

Edible food packaging systems are witnessing increased demand and hence, the market for sustainable foods is growing parallel to it. With this backdrop, it is imperative to develop innovative active food packaging that is sustainable and offers solutions to complex problems of the food industry, the most urgent one being that of food-borne antimicrobial resistance. Incorporating NAMAs into edible films gives us a better chance at food safety and preservation [102]. Although NAMAs added edible films rejoice in a special status among the research fraternity, they are yet to gain popularity in the industrial sector. The reason for the same is the poor adaptability of films in the commercial sector. Hence, further research is required to tailor the developed technology to suit the complex needs of the food packaging industry. The use of nanotechnology to develop encapsulated edible films with unvarying ultra-structure and improved barrier properties is one way to go about it. NAMAs added edible films hold a ticket to the bright future of edible films. However, apposite safety measures and toxicology studies need to be conducted before uptake of these agents in the food chain.

The mechanical and physical properties of the films should be improved upon by using specific additives that cater to the need of the food product or the end-use by the consumer [70]. NAMAs based edible films eliminate the need for synthetic preservatives to comply with the consumer demand for organic food products, encouraging a sustainable and healthy lifestyle [139]. The current demand patterns suggest that there will be ample market and high acceptability for active packaging that has a low carbon footprint and endorses sustainable consumption. Hence, future investigations should keep these facts in mind while developing modern NAMAs based edible films for potential use in the food industry.

## 9. Conclusions

This review explains the role of natural antimicrobials as additives in edible food packaging systems along with the sources of NAMAs, techniques of film formation and properties of films so formed. NAMAs enriched films can serve as an effective, non-toxic and sustainable alternative to the problematic plastic-based packaging. The increasing demand for environment-friendly solutions by the informed consumer slowly drives the food industry to a greener and cleaner approach. NAMAs have a lot to offer in terms of their beneficial physical and chemical properties to improve the quality of the foods they contain and the indirect biological effects upon entry into the human body. Ample literature is available on the laboratory scale NAMA-based films, but fewer studies are available on the industry scale films based on this technology. Hence, further studies on the commercial viability of NAMAs based edible films are recommended.

## Figures and Tables

**Table 2:** Food applications of edible films incorporated with NAMAs.

Food Product	NAMAs	Film Matrix	Preparation Method	Findings	References
**Fruits and Vegetables**					
Tomatoes and amla	Essential oil of turmeric/ginger/clove	Chitosan-based films	Heating & drying	Coated tomatoes and amla remained fresh for a longer time as compared to non coated samples	[85]
Figs	Cinnamon essential oil	Chitosan	Spread	The color change was delayed and *Alternaria alternata* growth was inhibited	[86]
Mangoes	Aloe vera	Chitosan-aloe vera films	Dipping	Suppress microbial decay (effective against Colletotrichumgloeospo-rides) of mango extend the storage life of mango fruit.	[87]
Dates	Pomegranate peel extract Citrus essential oils	Chitosan nanoparticles Chitosan and locust Bean Gum	Spreading Coating	Antifungal activity against mycotoxigenic fungi, *Aspergillus flavus*, *Aspergillus ochraceus and Fusarium moniliforme*. Significantly reduced conidial germination and completely inhibited *Aspergillus flavus* growth in dates	[88,89]
Apple and strawberry Cold stored fresh-cut apples	Olive oil *Stevia rebaudiana*	Chitosan Chitosan	Spreading Spreading	Protected against microbial decay Exhibited superior antimicrobial activity against mesophilic and psychrophilic aerobic bacteria	[90,91]
Strawberries and raspberries	Green tea extract	Alginate-oleic acid	Spreading	Antiviral activity	[92]
Fresh-cut melons	Coarse and nano emulsions of citral	Chitosan/carboxymethyl cellulose (CMC) polysaccharides	Nanoemulsified coatings	Superior antimicrobial protection (up to a 5-log reduction) and significantly extended the product’s storability (up to 13 days).	[93]
Capsicum	Pomegranate peel extract	Alginate based coatings	Blending	Maintain the chlorophyll, ascorbic acid, firmness and color while inhibiting the growth of the fungal pathogen *Colletotrichumgloeosporioides* at 10 °C storage	[94]
Organic baby spinach	Carvacrol or cinnamaldehyde	Hibiscus, carrot and apple-based films.	Blending and casting	Exhibited antimicrobial activity without affecting sensory characteristics of baby spinach	[95]
**Meat & Meat Products**					
Meat	Essential oil of Turmeric/Ginger/clove	Chitosan based films	Heating & drying	The meat remained fresh for seven days without any change in color, texture, odor and form.	[85]
Beef	*Lactobacillus sakei*	whey protein films	Spreading	Exhibited inhibitory action against *E. coli* or *L. monocytogenes*.	[96]
Chicken	Cinnamon essential oil and silver-copper	Linear low-density polyethylene	Wrapping	Showed enhanced antimicrobial activity and shelf-life was also increased	[97,98,99,100]
Fresh chicken breast Fresh Chicken Breast Fillets Chicken breast fillets	Oriental mustard extract (allyl isothiocyanate) Nisin and oregano oil Nisin	ĸ-Carrageenan and chitosan Guar gum (GG) and isolated soy protein Chitosan	Dipping Coating Coating	Effectively inhibited growth of *Campylobacter jejuni and* shelf-life was extended Delayed the growth of *Pseudomonas* and *Salmonella*, increasing the product shelf life (9 days) compared to the control samples (6 days) Reducing the growth of *Salmonella* and *Staphylococcus aureus*	
Abalone	Bamboo leaf extract	Alginate	Spreading	Enhanced microbial safety	[101]
**Dairy Products**					
Sweet meat or Doda Burfi	Nisin and Natamycin	Corn starch	Spreading	Superior efficacy against *Bacillus cereus and Aspergillus niger*	[102]
Gouda cheese	Lysozyme	Whey protein concentrate film	Casting method	Inhibit microorganisms (Lactic acid bacteria, *Enterococcus, Coliform, E. coli, Salmonella, S. aureus,* and yeast/mold) both at the surface and inside the region of gouda cheese during ripening.	[103,104,105,106,107,108]
Paneer (Cottage cheese)	Cinnamon essential oil	Sodium alginate crosslinked with calcium	Spreading	Increased the shelf-life of paneer samples to 13 days from 5-6 days and showed antimicrobial activity	
Low-fat cheese	Oregano essential oil	Nanoemulsion containing essential oil and mandarin fiber	Coating	Exhibited antimicrobial activity against *S. aureus and* extended the shelf life of low-fat cut cheese.	
Cheese	Essential oils of *Laurus nobilis* and *Rosmarinus officinalis*	Zein nanofibers	Coating	excellent antibacterial property against *S. aureus* and *L. monocytogenes* even after 28 days of storage	
Kashar Cheese	Ginger essential oil	Sorbitol, whey protein isolate, alginate	Coating	Exhibited antimicrobial properties against *Escherichia coli O157:H7* and *Staphylococcus aureus*	
Fresh Kashar cheese	Lysozyme	Zein-Carnauba wax	Coating	Significantly reduced bacterial (*Listeria monocytogenes*) count	

**Table 3:** Potential advantages and pitfalls of NAMAs in edible packaging.

Potential Advantages	Pitfalls
Antimicrobial edible packaging has a higher area to volume ratio and hence more competent to reduce microorganisms. Edible in nature- can be consumed with food products without any adverse effects. Shelf life can be controlled. Non-toxic and safe Environment friendly and biodegradable In general, no interaction with food components enhances the organoleptic properties of food. Individual packaging of food products like mango, banana and strawberry is feasible. Compatible with different shapes and sizes also. Edible packaging with antimicrobials has demonstrated complemented optical, thermal, mechanical and physicochemical properties.	Antimicrobial edible films do not ensure food safety where unsanitary conditions during food handling are there. Exhibit poor mechanical characteristics. Hence, an additional synthetic packaging material will be required during food product distribution and storage. Production cost is high If hydrophilic constituents are selected for edible films during manufacturing, the films will exhibit poor moisture and water vapor barrier properties, thus defying the aim of ensuring safe food to consumers. Some essential oils used as NAMAs are under the GRAS category, while some are prohibited for cytotoxic effects, toxicological reasons, or allergenicity. Hence, regulatory aspect should be taken into consideration. Some essential oils, if not encapsulated, may lead to alterations in color or sensory characteristics of food. If the target pathogen has a very short lag phase, then the biopolymer, which slowly releases antimicrobial compounds, will be ineffective in controlling their growth. If the antimicrobial incorporated into edible packaging is congenial with the constituents of edible film, it may not get released. In contrast, it is has a conflicting approach it may be released very swiftly.

## Data Availability

Data is contained within the article.
